# ROLES OF LONELINESS, STRESS, AND RELIGIOSITY IN SUICIDE IDEATION IN A SAMPLE OF SUB-SAHARAN AFRICAN OLDER ADULTS

**DOI:** 10.1093/geroni/igae098.2555

**Published:** 2024-12-31

**Authors:** Genevieve Ebulum, John Eze, Obinna Ezeihuoma, Crystal Njoku, JohnBosco Chukwuorji

**Affiliations:** David Umahi Federal University of Health Sciences, Uburu, Ebonyi, Nigeria; University of Nigeria Nsukka, Nsukka, Enugu, Nigeria; University of Pittsburgh, Pittsburgh, Pennsylvania, United States; American University of Antigua College of Medicine, Osbourn, Saint John, Antigua and Barbuda; Michigan State University Flint, Flint, Michigan, United States

## Abstract

Suicidal behaviour in older adults is a fundamental public health problem globally and the highest suicide rates occur in the low- and middle-income countries (LMICs). However, there is limited research on suicidality among older adults especially in sub-Saharan Africa. We sought to find out whether loneliness, stress and religiosity would significantly predict suicide ideation among Nigerian older adults (N = 500). They completed a questionnaire form comprising the Beck Suicidal Ideation Scale, the UCLA Loneliness Scale – version 3, Perceived Stress Scale and Religiosity Scale. While including age, educational qualification, and number of children as covariates, regression results showed that loneliness (β =.23) and stress (β =.10) were positively associated with suicide ideation while religiosity (β = -.16) exhibited a negative association with suicide ideation. The models substantially improved with the addition of each predictor. This suggests that whereas suicide ideation could be exacerbated by loneliness and stress, more religious older adults were less likely to report suicidal ideation. Interventions aimed at managing and protecting the mental health of older adults during their transition to late adulthood should guard them against loneliness and buffer their resilience and coping strategies with the connectedness that religiosity offers.

